# Moving beyond the Tip of the Iceberg: DJ-1 Implications in Cancer Metabolism

**DOI:** 10.3390/cells11091432

**Published:** 2022-04-23

**Authors:** Erika Olivo, Marina La Chimia, Jessica Ceramella, Alessia Catalano, Ferdinando Chiaradonna, Maria Stefania Sinicropi, Giovanni Cuda, Domenico Iacopetta, Domenica Scumaci

**Affiliations:** 1 Research Center on Advanced Biochemistry and Molecular Biology, Department of Experimental and Clinical Medicine, Magna Græcia University of Catanzaro, 88100 Catanzaro, Italy; erika.olivo@unicz.it (E.O.); marina.lachimia@unicz.it (M.L.C.); cuda@unicz.it (G.C.); 2 Department of Pharmacy, Health and Nutritional Sciences, University of Calabria, 87036 Arcavacata di Rende, Italy; jessica.ceramella@unical.it (J.C.); s.sinicropi@unical.it (M.S.S.); 3 Department of Pharmacy-Drug Sciences, University of Bari “Aldo Moro”, 70126 Bari, Italy; alessia.catalano@uniba.it; 4 Department of Biotechnology and Biosciences, University of Milano Bicocca, 20126 Milano, Italy; ferdinando.chiaradonna@unimib.it

**Keywords:** DJ-1, PARK7, cancer metabolism, ferroptosis

## Abstract

DJ-1, also called Parkinson’s protein 7 (PARK7), is ubiquitously expressed and plays multiple actions in different physiological and, especially, pathophysiological processes, as evidenced by its identification in neurodegenerative diseases and its high expression in different types of cancer. To date, the exact activity of DJ-1 in carcinogenesis has not been fully elucidated, however several recent studies disclosed its involvement in regulating fundamental pathways involved in cancer onset, development, and metastatization. At this purpose, we have dissected the role of DJ-1 in maintaining the transformed phenotype, survival, drug resistance, metastasis formation, and differentiation in cancer cells. Moreover, we have discussed the role of DJ-1 in controlling the redox status in cancer cells, along with the ability to attenuate reactive oxygen species (ROS)-dependent cell death, as well as to mediate ferropotosis. Finally, a mention to the development of therapeutic strategies targeting DJ-1 has been done. We have reported the most recent studies, aiming to shed light on the role played by DJ-1 in different cancer aspects and create the foundation for moving beyond the tip of the iceberg.

## 1. DJ-1: General Overview

The protein/nucleic acid deglycase DJ-1 (DJ-1, also called PARK7, Parkinson’s protein 7) is a highly conserved protein of 20 kDa, belonging to the DJ-1/ThiJ/Pfp protein superfamily [1], whose gene is located in the distal part of chromosome 1 (1p36.12–1p36.33) [2].

DJ-1 is a multifunctional protein that is ubiquitously expressed and acts primarily as a cysteine protease, although its functions range from a redox-regulating chaperone to a transcriptional co-activator, switching to a deglycase enzyme in a peculiar cancer context. Therefore, this protein appears to be involved in numerous physiological and pathophysiological processes, such as apoptosis, gene transcription, oxidative stress response, cell proliferation, and growth [3,4].

DJ-1 is predominantly a chaperone protein involved in redox homeostasis. The protein owns three cysteines (Cys) residues (Cys46, Cys53, and Cys106) essential for antioxidant proprieties. Among these residues, Cys106 is the most critical in maintaining the activity of redox sensor. Under hyper oxidative conditions, Cys106 might undergo oxidation eliciting the loss of antioxidant activity [5]. High levels of oxidized Cys106-DJ-1 have been associated with several pathologies, whose oxidative stress is the driving force in the physiopathology onset [3,4,6].

The conserved Cys106 region in the DJ-1 can be oxidized, inducing DJ-1 translocation into the mitochondria and repression of p53-dependent gene transcription. This region, necessary for DJ-1 homodimerization, has been reported to be the target of a recent series of bis-isatin derivatives that possess anticancer properties [6].

Mutations of the DJ-1 gene lead to a protein’s instability and loss of function, which is ultimately responsible for the death of dopaminergic neurons and the early onset of Parkinson’s disease [7].

Interestingly some of these mutations occur also on the region codifying for cysteine residue, especially Cys106, highlighting the crucial role of this residue in protein function and stability [8,9].

## 2. DJ-1 Status in Human Cancers

In addition to the widely known evidence for the role of DJ-1 in neurodegenerative diseases, several studies point to DJ-1 as a master regulator of neoplastic transformation [10]. This notion is supported by different studies. DJ-1 gene maps in a chromosomal locus, in which several chromosome abnormalities in cancer cells have been reported [2]. DJ-1 over-expression promotes, alone or in combination with other oncogenes (i.e., Ras and Myc), NIH3T3 cell transformation [11]. DJ-1 is highly expressed in many cancers with poorer prognosis, including breast, lung, pancreatic, thyroid, brain, and endometrial as well as different types of leukaemia [1,12,13,14,15].

From a mechanistic point of view, although the exact activity of DJ-1 in carcinogenesis has not been yet fully elucidated, it seems to be tightly associated with its ability to prevent oxidative damage and modulate peculiar cellular processes, such as signal transduction, apoptosis, invasion, and chemoresistance through the regulation of some key proteins such as tensin homolog (PTEN), mitogen-activated protein kinase (MAPK), nuclear factor kappa-light-chain-enhancer of activated B cells (NF-kB), Hypoxia-inducible factor 1-alpha (HIF-1α), androgen receptor (AR), and NF-E2–related factor 2 (NRF2) [16,17].

## 3. DJ-1 in Cancer Signaling

As previously anticipated, DJ-1 is considered an oncogene, mostly in association with other oncogenes such as c-Myc or H-Ras, and it can act, for instance, as a PTEN repressor, causing cell proliferation in both primary breast cancer cells as well as non-small cell lung cancer cells [18,19]. The ameliorated understanding of cancer onset and progression, thanks to the advances in genomic and proteomic technologies, have allowed for the genetic determination of the individual cancer risk. Moreover, in-depth genetic analysis disclosed an increasing number of known oncogenes and tumor suppressors in humans, whose combinations account for a million different cancer genotypes, together with the different single nucleotide variants in tumor cells compared to the normal ones [20,21].

However, there is disagreement about the role of DJ-1 in cancerogenesis, mainly because of the pleiotropic functions it exerts. For instance, DJ-1 is involved in transcriptional regulation [22,23,24], oxidative stress response [10], mitochondrial regulation [25], inflammation [26], and glycation damage prevention [27]. 

Several studies reported the requirement of DJ-1 for the maintenance of the transformed phenotype (e.g., uncontrolled proliferation, contact inhibition loss, anchorage-independent growth, extracellular matrix invasion) in cancer cells, as well as for the regulation of transformed growth, survival, chemoresistance, and metastasis formation and differentiation [28,29,30,31,32]. For instance, in the context of transcriptional activity, even though DJ-1 does not bind the DNA, it works as a transcription activator by sequestering inhibitory factors of crucial genes involved in cancer progression including p53, the AR, Nrf2, and PSF [33]. In addition, DJ-1 silencing by small interfering RNA (siRNA) inhibits cell transformation, cancer cell growth, and increases sensitivity to various chemotherapeutics [34,35,36,37]. Conversely, high DJ-1 activity induces the resistance of cancer cells to chemotherapy [28]. Such effect is due to its redox-sensitive chaperone activity, which participates in protecting cancer cells from oxidative agents, including some anticancer drugs [38,39]. Under oxidative conditions, indeed, oxidized DJ-1 is mainly localized into mitochondria, where it is able to protect cells from ROS-dependent damage [40], most likely by increasing the expression of antioxidant proteins, including glutamate cysteine ligase, and glutathione synthesis [41]. Altogether, these data suggest a relevant role of DJ-1 in cell transformation and chemoresistance, both most likely due to its function in maintaining mitochondrial integrity and in mediating protection from chemotherapy-induced oxidative stress.

## 4. DJ-1 Interplay with PI3K Signaling

DJ-1 modulates a classical survival pathway in some cancer cells, namely phosphatidylinositol 3-kinase/protein kinase B (PI3K)/(AKT), by suppressing the activity of the tumor suppressor gene phosphatase (PP2A) and PTEN, in turn promoting higher phosphorylation of PKB/Akt, a downstream target of PTEN, thereby supporting the transformation process and cancer cell proliferation and survival [42]. Several studies supported the idea that DJ-1 plays a negative regulation of PTEN, since when DJ-1 is overexpressed, as in urothelial carcinoma, lung cancer and melanoma, the expression of PTEN is reduced [43,44,45]. Conversely, DJ-1 silencing, for instance in human melanoma cells G361, causes upregulation of PTEN and some pro-apoptotic proteins, inhibition of AKT activity and anti-apoptotic proteins and thereby regulating their proliferation and invasion ability [46]. A similar effect has been reported in two models of papillary thyroid carcinoma, K1 and TPC-1 cells, where the knockdown of DJ-1 blocks proliferation, invasion, and migration through an increased PTEN expression and a decreased AKT phosphorylation. DJ-1 knock-down also reduces NF-κB activity and ERK1/2 phosphorylation, causing cell proliferation, migration, and invasion inhibition [17]. In NIH3T3 cells, DJ-1 hampers PTEN-induced apoptosis through activation of the PI3K/AKT pathway, glycogen synthase kinase 3 beta (GSK3β) phosphorylation, and cyclin D1 expression [42]. Similarly, in patients with high grade and poor prognosis glioma, DJ-1 expression has been associated with a higher level of protein catenin beta 1 (CTNNB1) [47]. Silencing DJ-1 in human hepatocellular carcinoma cells (HCCs) induces PTEN expression as well as inhibition of interleukin (IL)-6/Signal Transducer and Activator of Transcription 3 (STAT3), MAPK and AKT, resulting in reduced cell proliferation [48,49]. In leukemia cells, the DJ-1 knock out regulates the cell cycle via Cdk2, cyclin D1, c-Myc, NF-kB, Bcl-2, and PTEN, causing cell apoptosis [37]. Thus, it is clear that the survival of cancer cells is granted by the regulation of the activation status of different targets, triggered by DJ-1. 

## 5. DJ-1 Modulates the MAPK Signaling

DJ-1 prevents cell death and induces cancer cell invasion/migration by modulating the MAPK signaling pathway, which transmits signals from the cell membrane to the nucleus and regulates several cellular processes involved in oncogenesis [50]. Specifically, DJ-1 was found to be able to sequester the death protein Daxx in the nucleus causing the inactivation of the cytoplasmic stress-responsive effector apoptosis signal-regulating kinase 1 (ASK1), whose role is to activate JNK and p38, both promoting cell death [51,52]. Again, DJ-1 protects cells from UV-induced death through the mitogen-activated protein kinase/extracellular signal-regulated kinase 1 (MEKK1)-SEK1-JNK1 pathway. Indeed, DJ-1, by physically binding MEKK1, sequesters this kinase into the cytoplasm, and suppresses the downstream activation of SEK1 and JNK1 [53].

DJ-1 also plays a crucial role in cancer cell migration and invasion, as observed in pancreatic cancer cells, where it can activate the ERK/SRC phosphorylation cascade [54,55]. Several data report that DJ-1 is also physically associated with p53, a tumor suppressor that is mutated in almost half of human tumors and able to induce cell cycle arrest and apoptosis through the regulation of IGF-BP3 and/or PTEN gene expression, both involved in IGF-1/AKT pathway down-regulation [22,24]. Thus, the anti-apoptotic function of DJ-1 is explained by the suppression of the transcriptional activity of p53 through direct binding to the C-terminus of p53; consequently, p53 cannot activate the transcription of the pro-apoptotic factor Bcl-2 associated X (Bax), inhibiting the downstream caspase activation [23]. Finally, the association of DJ-1 expression with p53 appears to play a central role in determining the cell fate by regulating apoptosis [56]. Again, the regulation mediated by DJ-1 is pivotal in protecting cancer cells from death.

## 6. DJ-1 Implications in Hypoxia

Regulation of the PI3K/Akt/mTOR pathway by DJ-1 is also responsible for stabilizing the subunits of another important transcription factor, HIF-1, under hypoxia, which is essential for tumor progression [57]. The activation of insulin-like growth factor receptor (IGFR) or epidermal growth factor receptor (EGFR), promotes PI3K)/AKT signal transduction pathway, leading to an increased HIF-1α expression that, under non-hypoxic conditions, is rapidly ubiquitinated and degraded by proteasome [50]. Therefore, the total HIF-1 activity relies on HIF-1α protein levels [58].

In addition, DJ-1 expression, during hypoxia, protects cells from apoptosis by inhibiting caspase-3 cleavage activity [59]. Caspase-3 is normally activated during apoptosis, regardless of the death initiating stimulus, and is responsible of the cleavage of a wide array of substrates [60]. These events are associated with DJ-1 and contribute to cancer cell survival under hypoxic stress. 

Zeng et al. showed that DJ-1 promotes cell survival through the PI3K/AKT pathway and HIF-1α in human colorectal cancer (CRC) [61]. DJ-1 was found in 68.5% of these samples, with a higher nuclear localization compared to normal epithelial cells and associated with the tumor size and higher clinical stage. Importantly, DJ-1 levels were significantly associated with a high HIF-1α expression, which was identified in 74.0% of CRC samples. The authors suggest that DJ-1 regulates the PI3K/Akt/HIF-1α cell survival pathway and increases the expression of HIF-1α and other hypoxic genes. Worth mentioning is that HIF-1α is also activated by DJ-1 under non-hypoxic conditions [61]. These studies confirm that DJ-1 plays an important role in allowing cancer cells to escape from the hypoxic condition limitations. 

## 7. DJ-1 Regulates the Metastatic Process

Longhao et al. [62], using RNA sequencing and bioinformatics analyses, demonstrated that DJ-1 promotes epithelial to mesenchymal transition (EMT) in CRC cells through the Wnt signaling pathway. The DJ-1/Wnt pathway regulates the expression of fibroblast growth factor 9 (FGF9), which more highly expressed in CRC human samples, associated with tumor differentiation, poorer overall survival, and is closely correlated with other EMT markers such as E-cadherin and vimentin expression. Higher expression of DJ-1 associated with dysregulated levels of EMT biomarkers, particularly E-cadherin and vimentin, in esophageal squamous cell carcinoma (ESCC) tissue samples has been also reported [16] In human ECA-109 cells in vitro and in the in vivo nude mouse abdominal transplant model, DJ-1 overexpression is strongly associated with proliferation, migration, invasion, and EMT, mainly through the Wnt/β-catenin pathway [16]. These aspects should be taken into account for the development of drugs interfering with the metastatization process induced by DJ-1.

## 8. DJ-1 Regulates the Non-Canonical NF-κB Pathway

Recently, Shu et al. reported that DJ-1 is abnormally expressed in an endometrial cancer sample and is closely associated with the degree of differentiation, metastasis, and invasion [63]. Silencing of DJ-1 in Ishikawa cells causes cell viability inhibition and promotes apoptosis. As suggested by Zhu et al. [64], these events are associated with inhibition of the cellular zinc finger anti-NF-κB (Cezanne or OTUD7B) and subsequent activation of the non-canonical NF-κB pathway. Inhibition of Cezanne enables tumor survival through increased expression of IL-8 and ICAM-1 [65]. The authors demonstrated that Cezanne silencing reverts DJ-1 knockdown-induced proliferation inhibition. On the contrary, inhibition of the non-canonical NF-κB pathway by knocking down NF-κB-inducing kinase (NIK) abrogated the positive and negative DJ-1 effects on proliferation and apoptosis, respectively. Moreover, the authors showed that DJ-1 regulates NF-kB nuclear localization by directly inhibiting Cezanne. Altogether, these data suggest that Cezanne inhibition and non-canonical NF-κB signaling activation are involved in the ability of DJ-1 to regulate proliferation of endometrial cancer cells. 

## 9. DJ-1 Interactions with the Androgen Receptor

Several findings regarding DJ-1 activity have highlighted that the regulation of the AR signaling pathway is another important DJ-1 role, and a useful biomarker for several cancer types, including prostate cancer [66]. In this context, the expression of AR is greatly increased and its mutation, especially under treatment with AR antagonists, causes constitutive AR transactivation [67]. Importantly, treatments with androgens, such as dihydrotestosterone (DHT), or antiandrogens, such as OH-flutamide and bicalutamide, induce the translocation of DJ-1 into the nucleus. Meanwhile DJ-1 function in prostate cancer has not yet been fully elucidated, as other data indicated that DJ-1 expression increases the growth of the prostate cancer in patients treated with the androgen deprivation therapy [68]. DJ-1 can transcriptionally activate AR by forming a complex with the EF-hand calcium binding domain 6 (EFCAB6) and interfering with the association of EFCAB6 with histone deacetylase (HDAC) [69].

Moreover, DJ-1 interacts with the AR-binding region of the Protein Inhibitor of Activated STAT 2 (PIAS2), hampering the formation of the PIAS2/AR complex [70]. Qin et al. [71] suggested that DJ-1 overexpression induces survival of prostate cancer. In particular, they show that in LNCap prostate cancer cells, DJ-1, by inhibiting JNK and Bcl2 phosphorylation as well as Beclin1 and Bcl2 dissociation, causes a reduction of microtubule-associated proteins 1A/1B light chain 3B, namely LC3 (MAP1LC3B) and of auto-phagosome formation. Thus, the inhibition of autophagy due to both the DJ-1 and AR expression, in association with the growth of prostatic cancer cells, further strengthen the notion of a strict inter-regulation between DJ-1 and AR.

An association between DJ-1 expression and chemotherapy resistance has been observed in two gastric cancer cell lines. In particular, Liu et al. revealed that vincristine (VCR)-induced gastric cancer multi drug resistant (MDR) cells, SGC7901/VCR, as compared to a sensitive one, SGC7901, showed a higher level of DJ-1 associated with an increased survival and resistance to several other chemotherapeutics such as Adriamycin, 5-Fluorouracil, and Cisplatin, a phenomenon that was ascribed to the upregulation of P-gp and Bcl-2 [3]. 

## 10. DJ-1 and the Redox Homeostasis

DJ-1 is principally an antioxidant protein, controlling the redox balance in cancer cells, and is capable of protecting them from a ROS-induced cell death [51] through the transcriptional regulation of detoxification enzymes, including NAD (P)H-quinone oxidoreductase 1 (NQO1) [10]. However, further studies are needed to shed light on this aspect, as this deregulation appears to be cell type specific. Cao et al. reported that DJ-1 can block lipid peroxidation, protecting cancer cells from oxidative damage, and that its silencing reduces the intracellular reduced glutathione (GSH) levels. In addition, DJ-1 silencing or overexpression decreases or increases respectively the activity of SAHH (S-adenosyl-homocysteine hydrolase), the enzyme responsible for the catalytic production of homocysteine (Hcy) from SAH (S-adenosyl-L-homocysteine). The latter enzyme is involved in DJ-1-mediated ferroptosis, as its overexpression can reverse this effect [72]. Ferroptosis is a form of structured cell death characterised by a lethal increase of lipid hydroperoxides in response to iron high levels and increased intracellular ROS [73].

Ferroptosis is implicated in a variety of pathological contexts (e.g., Alzheimer’s, Huntington’s and Parkinson’s diseases, carcinogenesis, and stroke). It is known that certain degenerative diseases are triggered by an impaired ability to fight lipid peroxidation, leading to cell death. In addition, several authors propose ferroptosis as a scavenging mechanism for damaged cells that have been compromised by infection or environmental stress. 

Iron homeostasis and ROS metabolism are key players in the activation and regulation of ferroptosis [74,75]. Indeed, during oxidative stress, high levels of iron can promote ferroptosis through the Fenton reaction [76,77,78,79].

Ferroptosis might be essentially targeted by three classes of drugs: (i) iron chelators that influence the levels of iron; (ii) antioxidant agents, as lipophilic antioxidants and inhibitors of lipid peroxidation, that preserve cellular redox status; and (iii) molecule interfering with GSH metabolism [76,80,81,82,83,84,85,86,87,88,89,90,91,92,93,94]. All these molecules are categorized as “ferroptosis modulators” [95,96]. 

In this context, it is very tempting to dissect the role of DJ-1 as a peculiar ferroptosis inducer with the intent to shed light on a promising therapeutic target for cancer therapy. As previously discussed, DJ-1 directly counteracts oxidative stress undergoing oxidization at the Cys106 residue. In addition, it transcriptionally prevents ferroptosis modulating the expression of genes involved in lipid, ROS and iron metabolism. Furthermore, DJ-1 can stabilize Nrf2, the crucial transcriptional regulator of the antioxidant response [10,72,97]. Nrf2 is involved in the regulation of ferroptosis, cancer progression, invasion [98], and into resistance to therapy [99]. After stabilization, Nrf2 promotes iron storage, reduces cellular iron uptake, and limits ROS production.

Cellular redox status, resulting from the activity of endogenous catalytic activities, exogenous ROS inducer, and intracellular ROS scavengers, are essential for correct proliferation and differentiation [100,101,102,103]. 

In healthy conditions, Nrf2 has a pivotal role in cells survival, when oxidative stress is moderate, as it induces the expression of ROS scavengers and preserves cellular integrity [104]; conversely, when ROS levels are markedly increased, Nrf2 might trigger a plethora of programmed cell death types, including ferroptosis [91,105,106]. During cancer transformation, Nrf2 gains a pro-survival role, becoming a key factor in inflammation and resistance to therapy, and, accordingly, it is highly expressed in several types of cancers [107].

Recent findings suggest that NRF2 elicits cancerogenesis and tumour progression by inducing metabolic rewiring pathways and counteracting, also in tumour cells, the oxidative stress. This activity antagonizes the efficiency of chemotherapy and radiotherapy and fuels an ideal surrounding microenvironment for tumour cell growth [108].

In this scenario, establishing the ability of DJ-1 to direct Nrf2 activities might represent a valuable opportunity in cancer therapy.

**Figure 1:**
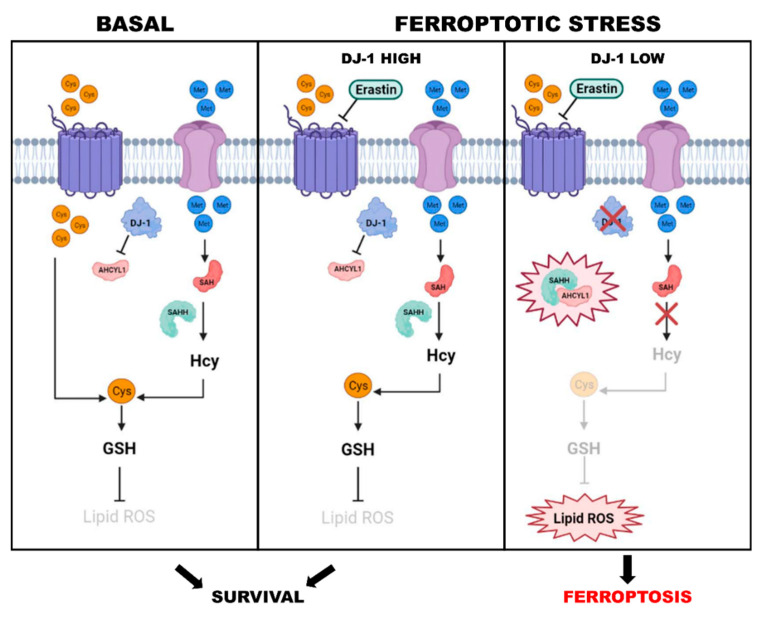
Role of DJ-1 in ferroptotic cells. In basal conditions, the intake of cystine is the driving force for GSH generation. During ferroptotic stress, DJ-1 level is a determining factor in the survival of the cells. In Erastin treated cells, the high levels of DJ-1 preserves the survival of cells by inducing the transsulfuration pathway. Conversely, low levels of DJ-1 inhibit the transsulfuration pathway, decrease the generation of GSH, and induce ferroptosis.

The role of DJ-1 in modulating Nrf2 activity and consequently ferroptosis is dual: directly, it promotes Nrf2 expression by preventing its ubiquitination and degradation [101]; indirectly, it acts as a reservoir of reduced groups for GSH biosynthesis by maintaining cysteine in the reduced thiol/thiolate state in the trans-sulfuration pathway. This evidence is remarkably supported by the finding that the sensitivity of various cancer cells to Erastin or analogues is greatly increased by DJ-1 suppression both in vitro and in vivo (Figure 1) [72,79,97,109]. Finally, in non-small cell lung cancer (NSCLC) cells, DJ-1 promotes cell proliferation. Remarkably, through its binding to the BH1-3 domain of Bcl-2-like protein 1 (BCL2L1), it is able to increase BCL2L1 mitochondrial stability, counteracting the antiproliferative effects of some oxidative agents such as ultraviolet B (UVB) radiation [110]. From these results it is evident that, under the ferroptotic stress, the DJ-1 levels play a major role in the survival of cancer cells.

## 11. DJ-1 Deglycase Activity

Increasing evidence suggests that DJ-1 and ROS are also associated with molecular pathways leading the formation of advanced glycation end products (AGEs). Under dicarbonylic stress, proteins and lipids undergo glycation, a non-enzymatic reaction that leads to the formation of methylglyoxal (MGO) and glyoxal (GO) products. The adducts formed through the Maillard reaction under oxidative conditions might be rearranged by producing AGEs [111], a damaging condition related to aging and senescence processes [112].

Although it is well established that ROS are the driving force leading to AGEs formation, it is also clear that AGEs are themselves a source of oxidative stress able to impair antioxidant scavengers. AGEs formation is thus a “self-feeding” mechanism correlated with ageing, cancer, neurodegeneration, and auto-immune diseases [113]. 

In the context of AGEs formation, DJ-1 is likely to have a leading role, being a glyoxalase able to revert the Maillard reaction [114]. In healthy conditions, the removal of dicarbonylic adducts is critical to avoid severe diseases [112,113] but, on the other hand, in cancer cells, this glyoxalase activity is crucial for preserving the replicative potential of cancers cell. 

The latest studies report that the aggressive malignancy unveils a hyper-glycolytic phenotype. This high glycolytic flux accounts for the formation of waste carbonylic species able to bypass nuclear membranes and react with the ε-amino groups of lysine and arginine, very represented in histone tails. This reaction, being non-enzymatic, is influenced only by the levels of reagents and is thus dependent on glycolytic flux.

Glycation might induce the deconstruction of histone codes impairing the replicative cellular potential. Based on these observations, high glycolytic cancer cells should be more susceptible to senescence but, paradoxically, this type of malignancy exhibits a more aggressive phenotype. The reason lies in the overexpression of DJ-1 that counteracts AGEs formation both by reverting glycation reaction and by reducing oxidative stress. 

**Figure 2:**
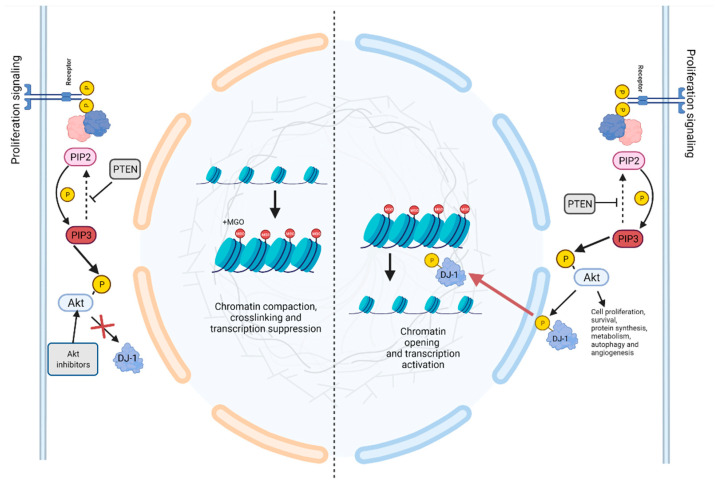
Role of DJ-1 during dicarbonyl stress. In senescent cells, the hyper-glycolitic phenotype induces dicarbonyl stress. Left side: In cells treated with Akt inhibitor, DJ-1 does not undergo phosphorylation. Unphosphorylated DJ-1 loses its glyoxalase activity and is unable to counteract the formation of MGO adducts, accounting for chromatin destructuration. Right side: In proliferating cell, under the activation of the Akt pathway, DJ-1 undergoes phosphorylation and translocates into the nucleus where it acts as glyoxalase. This activity preserves the histones code and the malignant proliferative potential.

In this context, two key papers concomitantly highlighted the role of DJ-1 in preserving nucleosome stability and chromatin architecture, by removing MGO and GO adducts from histone tails [115,116]. The authors depicted a novel molecular mechanism, connecting the cellular metabolic alteration with the epigenetic perturbation. Notably, they emphasized that high levels of glycation correlate with DJ-1 overexpression, enforcing the notion that DJ-1 has a strategic role in preserving the malignant replicative potential of breast cancer cells. On the other hand, our research group deepened this topic, adding a further order of complexity to the molecular mechanism. We proposed that DJ-1 might undergo Akt-dependent phosphorylation on the catalytic site, providing the basis to demonstrate that the peculiar mitogenic signal, as the activation of PI3K pathway, might direct DJ-1 deglycase activity (Figure 2). Our work suggests that in cancer cells, the peculiar DJ-1 proteoform accounts for its role in epigenetic misregulation preserving the histone code and promoting survival [117].

## 12. The Interplay between DJ-1 and miRNAs in Cancer and Oxidative Stress Related Conditions

A number of recent studies have functionally linked DJ-1 to some microRNAs, which are crucial post-transcriptional regulators. This interplay seems to be involved in the development and progression of several unhealthy conditions, including cancer and Parkinson’s disease. Concerning cancer, several DJ1-miRNAs interactions have been described. In particular, miR-216b inhibits gastric cancer proliferation and migration by targeting DJ-1, latter promoting gastric cancer peritoneal metastasis through PI3K/Akt signalling pathway [118]. Interestingly, modulation of DJ-1 levels by miRNAs has also a crucial role in overcoming chemoresistance. For instance, in pancreatic cancer cells and in HCC, MiR-203 affects cisplatin resistance by inducing apoptosis [119]. Furthermore, MiR-128-3p overexpression sensitizes cancer cells to sorafenib-induced apoptosis [120]. 

Similarly in glioma, MiR-544 inhibits cells proliferation, invasion, and migration by targeting the DJ-1 gene [121]. The interplay between DJ-1 and miRNAs has also been described in several neurological diseases. For instance, in Parkinson’s, DJ-1 seems to modulate the expression of miR-221 promoting neuronal survival and hampering oxidative stress. Specifically, DJ-1 increases miR-221 expression through the MAPK/ERK pathway, leading to the repression of apoptotic effectors [122]. Conversely, two miRNAs, Hsa-miR-4639-5p and miRNA-494, down-regulating DJ-1, elicit severe oxidative stress and neurodegeneration [123,124]. In the context of degenerative disease, Xue and colleagues described how the overexpression of miR-122, leading to DJ-1′s downregulation, counteracts the ischemia-reperfusion damage after cerebral infarction [125]. Additionally, an interesting study reported that MiR-181a, regulating the expression of p62/SQSTM1, parkin, and DJ-1, promotes mitochondrial dynamics in skeletal muscle ageing. The study underlies that the age-related downregulation of MiR-181a is associated with the accumulation of autophagy-related proteins and abnormal mitochondria while the restoring of miRNA levels in old mice, prevents the accumulation of p62, DJ-1, and PARK2, and improves mitochondrial quality and muscle function [126]. Finally, it is reported that the downregulation of miR-4485-3p is associated with asthenozoospermia through the interaction with DNAH1, KIT, and PARK7 genes [127]. Overall, these studies highlight that DJ-1 exerts a dual role based on pathophysiological context. In cancer cells it exhibits a prosurvival role by directing proliferation, migration, invasion and chemoresistance through the modulation of mitogenic pathways; on the other hand, in neurological diseases, where it acts principally as an antioxidant agent, its downregulation might induce serious cellular damage by hampering redox homeostasis.

## 13. DJ-1 as a Potential Therapeutic Target

Taking into account the widespread functions of DJ-1, it is obvious that several research groups pointed out the development of therapeutic strategies aiming to target DJ-1. 

Some strategies blocking the biological functions of DJ-1 in cancer are based, for instance, on DJ-1 gene silencing or interference with the controlled pathways, but few studies regarding compounds able to bind directly DJ-1 have been published and the description of their precise mechanism of action is not yet detailed in depth [128]. The major reason is the lack of a potent and well-characterized chemical inhibitor, however some interesting studies, based on crystal structure and computational studies, have been conducted on compounds that bind the Cys106 region [129]. Bilsland et al. reported the identification and characterization of a pyrazolopyrimidine compound series (particularly, CRT0063465 and its analogue CRT0105481), even though the inhibition mechanism was not really clarified [129].

In the DJ-1 structure, the conserved Cys106 is the main residue that influences many known functions. The Cys106 region serves as a sensor of redox homeostasis and can be oxidized to both the sulfinate (-SO_2_^−^) and sulfonate (-SO_3_^−^) forms [130]. Oxidation of Cys106 seems to be crucial for DJ-1 translocation into the mitochondria, interaction with p53, and repression of p53-dependent gene transcription [11]. Thus, considering that this residue seems to play a key role in the modulation of DJ-1 activities, the docking of small molecular compounds able to target this region represents an exciting therapeutic strategy.

Tashiro and colleagues applied a fragment-based methodology to identify novel inhibitors of DJ-1, focusing on molecules capable of binding the Cys106 region. These promising compounds exhibit significant inhibitory properties in a cell-based assays, targeting both the redox sensor function and the DJ-1 deglycase activity [128]. 

Yanagida and co-workers identified a molecule (UCP0054278) capable of binding to the SO_2_H-oxidized Cys106 region. The molecule enhances DJ-1′s anti-oxidative and anti-apoptotic activity, preventing both ROS production and neuronal cell death [131].

Although oxidation of Cys106 residue is essential for the DJ-1 catalytic activity, it is extensively demonstrated that an excessive oxidation induces protein inactivation and is the driving force for Parkinson disease onset. In this context, two emblematic papers described a subset of molecules that exhibit the ability to specifically bind the DJ-1 Cys106 region maintaining its active forms. These molecules share the capability to prevent the excessive oxidation of DJ-1, inhibiting the oxidative induced cell death [132,133].

A recent work discusses the synthesis and the analysis of a series of bis-isatin derivatives able to specifically target the DJ-1 homodimerization unveiling remarkable anti-cancer properties [6]. Intriguingly, the authors describe a molecule (DM10) that significantly induces apoptosis and erastin-based ferroptosis in several human cancer cell lines and, much more interestingly, also in xenograft mice generated from H1299 cells. Finally, in the brilliant work by Maksimovic and colleagues, they developed a fluorescence-based assay to screen DJ-1 inhibitor exploring its esterase activity by DiFMUAc substrate. Using this approach, the authors enlarge the parterre of reversible and irreversible inhibitors of DJ-1 providing novel promising isatin-analog molecule able to impair DJ-1 activity [134].

## 14. Conclusions and Future Perspectives

**Figure 3:**
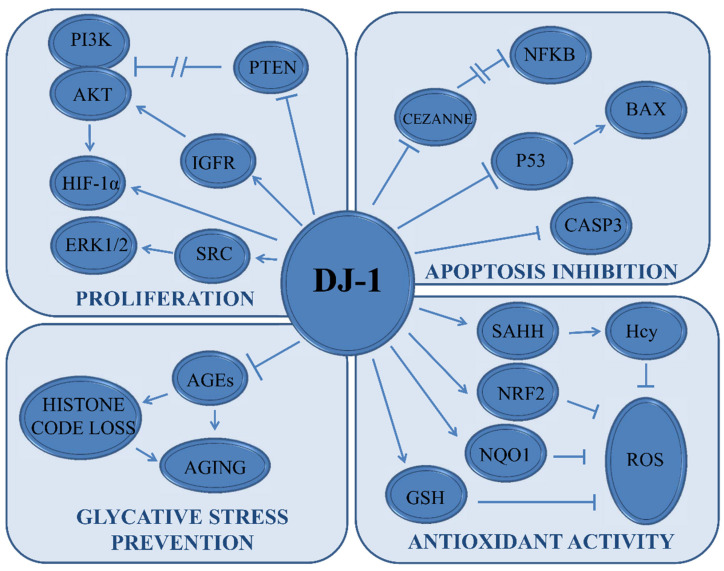
DJ-1 Downstream signaling. The scheme is a synopsis of DJ-1 downstream effectors. DJ-1 is involved in cell proliferation, Apoptosis evasion, redox homeostasis, and prevention of glycative stress. For each signaling pathways, the main effectors are reported. We reported the effects of DJ-1 on phosphatidylinositol-3 kinase (PI3K), protein kinase B (AKT), phosphatase and tensin homolog (PTEN), insulin-like growth factor receptor (IGFR), hypoxia-inducible factor 1-alpha (HIF-1α), extracellular signal-regulated kinase 1/2 (ERK1/2), tyrosine-protein kinase (SRC), nuclear factor kappa-light-chain-enhancer of activated B cells (NFKB), cellular zinc finger anti-NF-κB (CEZANNE), Bcl-2-associated X protein (BAX), (P53), caspase 3 (CASP3), advanced glycation end-products (AGEs), S-adenosyl-homocysteine hydrolase (SAHH), homocysteine (Hcy), nuclear factor erythroid 2-related factor 2 (NRF2), NAD(P)H-quinone oxidoreductase 1 (NQO1), glutathione (GSH), and reactive oxygen species (ROS).

Here we propose an analysis of DJ-1 function, pointing out that this mitochondrial protein exerts a crucial role in several physiological and pathological pathways (Figure 3). Closely, we dissect the activity of DJ-1 as a master regulator of cellular redox homeostasis, highlighting that this function is, in turn, strategic for the modulation of ferroptosis as well as glycation. Therefore, we propose a synopsis of the current strategies aiming to clarify the role of this protein in cancer (Table 1). 

Some effective approaches for regulating DJ-1 function in cancer have been published, mostly reporting the interference with its gene expression or pathways modulated by DJ-1. However, studies on the direct blockade of DJ-1 at the protein level are still controversial and need to go more in-depth. One of the major issues is represented by the lack of a potent and ascertained DJ-1 inhibitor. Some studies reported the interaction of a few compounds with the region responsible of DJ-1 homodimerization, demonstrating that its blockade induces cancer cells death. Unfortunately, these outcomes still have many unanswered questions regarding the ability of DJ-1 to form many complexes, its subcellular localization, and the adopted cell context. Thus, it is desirable to enlarge these results in other cancer types and under different experimental conditions, and, most importantly, the current research should focus on the individuation of the DJ-1 complexes and its subcellular interactions, the exact role under oxidative stress, and in chemoresistance.

Overall, our review aims to underlie that DJ-1 is functionally required for several aspects of the transformed phenotype and suggests that disrupting its protective activity may be a promising therapeutic approach in the fight against cancer. 

## Figures and Tables

**Table 1:** Correlating the expression and the regulation of DJ-1 with different types of cancers.

DJ-1 Expression	Regulation	Type of Cancer	Ref.
Overexpression	Reduction of PTEN expression	Urothelial carcinoma lung cancer	[43,44,45]
Silencing	Upregulation of PTEN and other pro-apoptotic proteins; inhibition of the activation of AKT and anti-apoptotic proteins	Human melanoma cells G361	[46]
Knock-down	Increased PTEN expression and decreased AKT phosphorylation	Papillary thyroid carcinoma, K1 and TPC-1 cells	[17]
Knock-down	NF-κB activity reduction and ERK1/2 phosphorylation	Papillary thyroid carcinoma, K1 and TPC-1 cells	[17]
Overexpression	Activation of the PI3K/AKT pathway, GSK3β phosphorylation and cyclin D1 expression	Transformed NIH-3T3 cells	[42]
Expression	Increasing CTNNB1 level	Patients with high grade and poor prognosis glioma	[47]
Silencing	Increased PTEN expression, inhibition of interleukin (IL)-6/Signal Transducer and Activator of STAT3, MAPK and AKT	Human hepatocellular carcinoma cells (HCCs)	[48,49]
Knock out	Regulation of Cdk2, cyclin D1, c-Myc, NF-kB, Bcl-2 and PTEN	Leukaemia cells	[37]
Expression	Activation of the ERK/SRC phosphorylation cascade	Pancreatic cancer cells	[54,55]
Expression	Regulation of PI3K/AKT pathway and HIF-1α	Human colorectal cancer (CRC)	[61]
Expression	Regulation of Wnt signaling pathway	CRC cells	[62]
Overexpression	Increased EMT process	Esophageal squamous cell carcinoma (ESCC) tissue samples	[16]
Overexpression	Wnt/β-catenin pathway, increased EMT process	Human ECA-109 cells in vitro and in the in vivo nude mouse abdominal transplant model	[16]
Silencing	Inhibition of the cellular zinc finger anti-NF-κB (Cezanne or OTUD7B)	Ishikawa cells	[64]
Overexpression	Inhibition of JNK, Bcl2 phosphorylation/dissociation, Beclin1	LNCap prostate cancer cells	[71]
Expression	Increased BCL2L1 mitochondrial stability	Non-small cell lung cancer (NSCLC) cells	[110]

## Data Availability

Not applicable.
